# High Potential for Biomass-Degrading Enzymes Revealed by Hot Spring Metagenomics

**DOI:** 10.3389/fmicb.2021.668238

**Published:** 2021-04-21

**Authors:** Nicholas J. Reichart, Robert M. Bowers, Tanja Woyke, Roland Hatzenpichler

**Affiliations:** ^1^Department of Chemistry and Biochemistry, Montana State University, Bozeman, MT, United States; ^2^Thermal Biology Institute, Montana State University, Bozeman, MT, United States; ^3^Center for Biofilm Engineering, Montana State University, Bozeman, MT, United States; ^4^Department of Energy, Joint Genome Institute, Berkeley, CA, United States

**Keywords:** bioprospecting, thermophilic enzymes, biotechnology, CAZyme, cellulases

## Abstract

Enzyme stability and activity at elevated temperatures are important aspects in biotechnological industries, such as the conversion of plant biomass into biofuels. In order to reduce the costs and increase the efficiency of biomass conversion, better enzymatic processing must be developed. Hot springs represent a treasure trove of underexplored microbiological and protein chemistry diversity. Herein, we conduct an exploratory study into the diversity of hot spring biomass-degrading potential. We describe the taxonomic diversity and carbohydrate active enzyme (CAZyme) coding potential in 71 publicly available metagenomic datasets from 58 globally distributed terrestrial geothermal features. Through taxonomic profiling, we detected a wide diversity of microbes unique to varying temperature and pH ranges. Biomass-degrading enzyme potential included all five classes of CAZymes and we described the presence or absence of genes encoding 19 glycosyl hydrolases hypothesized to be involved with cellulose, hemicellulose, and oligosaccharide degradation. Our results highlight hot springs as a promising system for the further discovery and development of thermo-stable biomass-degrading enzymes that can be applied toward generation of renewable biofuels. This study lays a foundation for future research to further investigate the functional diversity of hot spring biomass-degrading enzymes and their potential utility in biotechnological processing.

## Introduction

Lignocellulose is one of the most abundant polymers on Earth and is used in many industrial settings including textile processing, paper milling, and biofuel conversion (Cabrera and Blamey, 2018). Non-food crops and agricultural waste present a target for the conversion of lignocellulose into ethanol as a second-generation biofuel, without the need for competition with food sources, such as corn and wheat (Gupta and Verma, 2015). However, lignocellulose is extremely recalcitrant and difficult to break down in a cost-efficient manner (Cheah et al., 2020). In 2019, the United States of America consumed approximately 541 billion liters of gasoline while only producing 60 billion liters of bioethanol (Renewable Fuels Association website for production)^1^. Reducing the costs of biofuel production while increasing yield would allow renewable energy to become a greater portion of today’s energy market.

Lignocellulose is composed of lignin, hemicellulose, and cellulose in varying compositions depending on the plant source (Gupta and Verma, 2015; He et al., 2018). The lignin component contains aromatic compounds that encompass branched polysaccharides of hemicellulose and crystalline chains of cellulose. Currently, industrial processing of lignocellulose occurs with costly mechanical or chemical pretreatment to depolymerize the lignin component and make the saccharides more accessible (Cheah et al., 2020). Pretreatment using various methods increases the amount of saccharification by an order of magnitude over untreated biomass (Bala and Singh, 2019). Removal of lignin through pretreatment exposes hemicellulose and cellulose and increases surface area available for saccharification by continued pretreatment or enzymatic degradation. The released sugar products can then be fermented to produce biofuels.

Prior to fermentation, a multitude of enzymes are required for the complete degradation of lignocellulose, including laccases, hemicellulases, and cellulases. The Carbohydrate Active Enzyme (CAZy) database (Lombard et al., 2014) provides sequence-based classification families of the many types of enzymes involved. Lignin degradation occurs from laccases and peroxidases most commonly in the Auxiliary Activity (AA) families 1 and 2, respectively (Levasseur et al., 2014). These enzymes catalyze oxidation reactions of phenolic compounds and have been widely studied in fungi and some aerobic bacteria (Bugg et al., 2011). Hemicellulases, including xylanases, mannases, and other debranching enzymes, catalyze the hydrolysis of β-1,4 glycosidic bonds of the corresponding sugars and these enzymes are classified in a multitude of Glycosyl Hydrolase (GH) families. Cellulases act exclusively upon the Glucose-β-1,4-Glucose bonds of long chain fibrils that are encompassed by hemicellulose and lignin. As with hemicellulases, cellulases are classified as GHs and are either endo- or exo-acting, depending on the structure of their active site (Davies and Henrissat, 1995).

Lignocellulolytic enzymes and their activities have been studied in detail (Zarafeta et al., 2016; Zayulina et al., 2020), but the discovery of new enzymes has mainly been limited to isolated microorganisms or enrichment cultures. Most cellulolytic isolates come from only three classes of bacteria, *Actinobacteria*, *Bacilli*, and *Clostridia* (Bayer et al., 2013). Much of the early work on bacterial cellulases was performed with members of the genus *Caldicellulosiruptor* (originally *Anaerocellum*) and its multi-domain, high activity CelA (Brunecky et al., 2013; Young et al., 2014). Traditionally, enrichment cultures have been established using crystalline cellulose or plant biomass (e.g., corn stover) and have captured consortia of cellulolytic capabilities (Graham et al., 2011; Zhao et al., 2014). However, rare cellulolytic community members or members of yet uncultured lineages are often missed in these traditional assays and require novel techniques to be captured (Doud et al., 2019). *In silico* discovery of CAZymes has also been used to identify novel cellulolytic genes from metagenomes and the subsequent expression of the enzymes in recombinant hosts allowed for characterization studies to be conducted (Wohlgemuth et al., 2018; Strazzulli et al., 2020) and the activities could be tested under pretreatment conditions, such as ionic liquids (Heins et al., 2014).

Many studies have tested environmental samples and cellulolytic enrichments for microbial biomass degradation activity, but these studies have generally been limited to taxa that grow during the enrichment or are naturally abundant. Rumen (Hess et al., 2011; Henderson et al., 2015), termite guts (Marynowska et al., 2020), compost (Ma et al., 2020), forest soils (Wilhelm et al., 2018), and hot springs (Peacock et al., 2013) are sample types that have been the focal points of discovering yet uncultivated cellulolytic organisms. Hot springs are a particularly favorable system for further research into lignocellulolytic enzymes because of the enzymatic adaptations to the extreme conditions in these ecosystems. Industrial chemical pretreatment can range from pH 1 to 13 (Pedersen et al., 2011) with variable activity of the pretreatment processes toward the different components of lignocellulose depending on the pH of the system. Such extreme conditions are typically absent from most natural systems but are normal in geothermal systems where thermostable enzymes have been selected to be active across a broad range of temperature and pH. For example, sites in Yellowstone National Park (YNP) exhibit a wide range of pH (0.52–10.6) and temperature (10–99°C)^2^ for a database of ∼7,700 features in the park. Hot springs in New Zealand exhibit similar extreme conditions (pH < 1–9.7, 14–101°C; Power et al., 2018). Thermostability in lignocellulose processing is desired because it allows for increased substrate solubility, favorable enzyme kinetics, and lowers the risk of contamination by unwanted organisms. Exploring environmental metagenomes of microbial communities from thermal sites across a wide spectrum of temperature and pH provides a unique opportunity to expand the repertoire of known extremozymes with lignocellulolytic functions.

In this study we utilized 71 publicly available metagenomes from 58 unique geothermal features to analyze the microbial diversity and CAZyme composition in hot spring systems. To our knowledge, this is the largest survey of hot spring metagenomes for biomass degradation potential. We connected microbial populations to high lignocellulolytic potential based on temperature and pH of the hot springs to identify target springs for future biotechnology applications. This work provides an initial exploratory approach to mining metagenomic datasets of a wide range of physicochemical conditions in hot springs. The diversity of biomass-degrading potential described here will provide new targets for future *in vitro* functional assays of thermophilic CAZymes that can be tested using methods, such as synthetic biology in conjunction with functional assays.

## Materials and Methods

Assembled metagenomic samples were retrieved from the JGI’s Integrated Microbial Genomes and Microbiomes (IMG/M; Chen et al., 2021) database and followed the standard assembly and annotation protocol used for JGI-sequenced metagenome datasets at the time of submission. Corresponding assembly and annotation methods are listed in Supplementary Table 1 as provided by IMG/M. We acknowledge variation in these methods as a result of comparing datasets generated over a long range of time. Criteria for selection were as follows; Genomes OnLine Database (GOLD) (Mukherjee et al., 2021) analysis project type as metagenomic analysis and GOLD ecosystem type as thermal springs. Repeated metagenome hot springs sites were selected based on sequencing size or presence of a linked publication in GOLD. Several sites were included as unique samples corresponding to different transects from the source water (i.e., Dewar Creek) or due to temporal sampling (i.e., Jinze). Metadata for spring location, temperature, and pH were retrieved from IMG and GOLD (Supplementary Table 1). For missing data, the corresponding publications were searched, or the PIs were contacted for unpublished samples. PIs of all published and unpublished metagenome datasets were contacted to acquire permission of data usage; all PIs gave permission to use their data.

To assess the taxonomic profile, Kraken2 (Wood et al., 2019) and Bracken (Lu et al., 2017) were used to assign the assembled contigs taxonomy and calculate the percentage of each taxa per metagenome, respectively. Kraken2’s standard database was built using the –download-library switch with options bacteria and archaea. Each metagenome was run through Kraken2 using GNU parallel (Tange, 2018) and 8 threads per job using default k-mer length of 35. Bracken was used for estimation of taxa abundance from Kraken2 reports using options -r 150, -l P, and -t 10. Diversity metrics of the taxonomic profile were calculated using R (R Core Team, 2014) package Phyloseq and statistical analyses with lme4 (Bates et al., 2015) and vegan (Oksanen et al., 2019) packages.

Lignocellulolytic enzymes were identified from the Carbohydrate Active Enzyme Database (Lombard et al., 2014)^3^ using the dbCAN2 package (Zhang et al., 2018). The HMMER, HotPep, and DIAMOND tools were applied using default parameters to annotate CAZymes in the assembled metagenomic samples. Only CAZy gene hits that were present in at least two of the three tools were considered for later analysis. All computing was performed on the NERSC Cori cluster. The CAZyme heatmap was generated using R package Pheatmap (Kolde, 2015) with presence-absence normalized data from the decostand function from the vegan package and the heatmap was annotated with data available from IMG. An additional heatmap was generated for CAZyme relative abundance (Supplementary Figure 1). Four metagenomes were selected for deeper analysis. TaxonOID’s 3300029977, 3300006865, and 3300007072 were selected because of their high abundance of glycosyl hydrolases (GH). While 330009455 also had a similar number of GHs, we did not include this sample due to it having the lowest sample collection temperature (28.5°C). TaxonOID 3300029625 was included as the fourth sample because it contained a majority of the GHs but was also the closest clustered sample with acidic pH. The most abundant GH for each enzyme type was further described by selecting the gene identification number and searching the metagenome on IMG for the corresponding contig. Out of the three GHs (GH3, GH5, and GH10), the main catalytic Pfam domain was selected and those sequences were queried in IMG/M for taxonomic affiliation. The data containing enzyme class, taxonomic distribution, and metagenome location was illustrated in a Sankey diagram using R package networkD3 (Allaire et al., 2017).

## Results and Discussion

### Metagenomic Dataset

**FIGURE 1:**
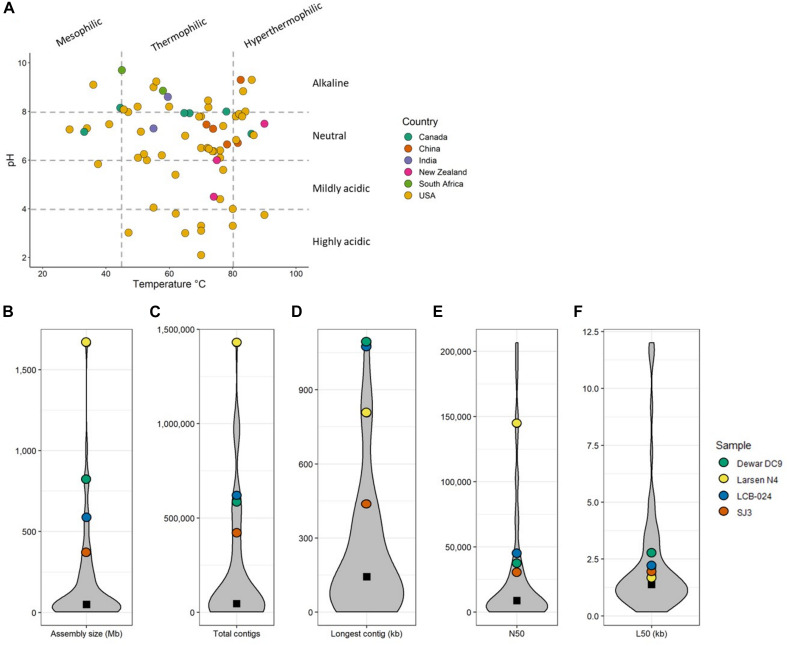
**(A)** Temperature and pH scatter plot of all 71 metagenomes used in this study. Points are colored by country of sample origin. Dashed gray lines denote the temperature and pH categories used. Temperature categories: mesophilic (<45.0°C), thermophilic (45.0–79.9°C), and hyperthermophilic (>80.0°C). pH categories: highly acidic (<4.0), mildly acidic (4.0–5.9), neutral (6.0–7.9), and alkaline (>8.0). **(B–F)** Violin plots of sequencing statistics for all 71 metagenomes. The four metagenomes of particular interest to this study are highlighted. Black square denotes median value.

Metadata filtering to retrieve hot spring metagenomes from the Integrated Microbial Genomes and Microbiomes system (IMG/M) (Chen et al., 2021) yielded 71 assembled metagenomic samples from 58 unique hot springs (Supplementary Table 1). Several sites had multiple samples included if distinct areas of the hot spring were sampled (e.g., Dewar Creek transects) or when sampling occurred in consecutive years (e.g., Jinze). Geothermal features were distributed across six countries, with the majority located in the United States (Figure 1); this is because of the long tradition of hot spring research in Yellowstone National Park (YNP), which hosts approximately 55% of the world’s geothermal features. The temperature and pH ranges of the samples were 28.5–90°C and 2.1–9.7, respectively (Figure 1). Metagenomic sequencing size varied considerably among samples with a median assembly size of 49.4 Mb. The wide range of assembly size (3.2–1,666.8 Mb) is attributed to the different sequencing technologies and facilities used. As IMG/M is a repository for metagenomic data, not all samples have been processed the same way prior to annotation. All metagenomic sequencing metadata of samples used in this study has been collected in Supplementary Table 1.

### Taxonomic Profile

Kraken2 (Wood et al., 2019) was used to assign taxonomy to the assembled contigs followed by processing with Bracken (Lu et al., 2017) to calculate the relative abundances of taxa in each metagenomic sample. We first categorized the metagenomic datasets based on temperature and pH to identify taxonomic trends due to these parameters. The temperature categories were mesophilic (<45.0°C), thermophilic (45.0–79.9°C), and hyperthermophilic (>80.0°C). The pH categories were highly acidic (<4.0), mildly acidic (4.0–5.9), neutral (6.0–7.9), and alkaline (>8.0). The description of sites within each category is summarized in Supplementary Table 1. Overall, at the phylum level, contigs were identified most frequently as being derived from members of *Proteobacteria*, followed by *Firmicutes*, *Actinobacteria*, *Aquificae*, and *Bacteroidetes*. Out of all contigs that could be classified using the Kraken2 standard database, 45 archaeal and bacterial phyla were identified in total across all metagenomes (Supplementary Table 2). *Proteobacteria* dominated mesophilic and thermophilic samples while *Aquificae* was the most abundant phylum in hyperthermophilic samples; the latter has been well-documented for high temperature streamer communities in YNP (Inskeep et al., 2013a,b). The phylum *Crenarchaeota* was also most abundant in highly acidic and mildly acidic samples, while *Proteobacteria* were the dominant phylum in neutral and alkaline samples. Microbes belonging to *Aquificae* and *Crenarchaeota* have been shown to be widespread in geothermal environments with genus-specific adaptations to narrow temperature or pH conditions (Hou et al., 2013). Phylum level taxonomy can highlight overall trends but to understand how microbial populations are distributed across temperature and pH conditions, finer taxonomic resolution is required.

Across all 71 metagenomes, the assembled contigs were assigned to 1,474 unique genera. In the mesophilic category, *Streptomyces* was the most abundant genus, closely followed by *Roseiflexus* and *Pseudomonas*. In thermophilic samples, there was a taxonomic shift toward *Thermus*, *Sulfurihydrogenibium*, *Thermocrinis*, and *Bacillus* assigned contigs. While contigs assigned to *Thermocrinis* were still in moderately low abundance in thermophilic samples, they were the top contig assignment for hyperthermophilic samples followed by *Pyrobaculum* and *Thermus*. Highly acidic metagenomes contained contigs assigned to *Hydrogenobaculum*, *Sulfolobus*, and *Acidolobus*, among others. Consistent with reports that *Hydrogenobaculum* is the dominant genus of *Aquificae* in thermophilic, acidic hot springs (55.1–64.5°C, pH 2.5–2.6; Hou et al., 2013; Takacs-Vesbach et al., 2013), members of this genus were abundant in metagenomes of the highly acidic category. Members of the crenarchaeotal genus *Sulfolobus* are common and abundant in acidic hot springs in both YNP and Chinese sites (Inskeep et al., 2010; Song et al., 2013).

Our results are largely consistent for most taxa with the findings of Inskeep et al. (2013a, b) who previously analyzed 20 unique features in YNP. However, our expanded dataset, which consisted of 58 unique features, including features outside YNP, included a higher abundance of *Sulfurihydrogenibium* in acidic sites than previously observed. In the mildly acidic range, *Acidilobus* was dominant, followed by *Streptomyces* which was more abundant than it was in highly acidic samples and remained abundant, although not dominant, in the metagenomes from pH-neutral sites. The pH-neutral samples were dominated by genera *Thermocrinis* and *Thermus*. A study of 16 Chinese hot springs detected high amounts of *Thermocrinis* and *Thermus* sequences in high temperature, non-acidic samples (Song et al., 2013); we observed similar trends for both taxa in similar temperature and pH categories. Finally, in alkaline features, metagenomes were taxonomically dominated by *Thermocrinis*-, *Bacillus*-, and *Thermus*-affiliated contigs.

**FIGURE 2:**
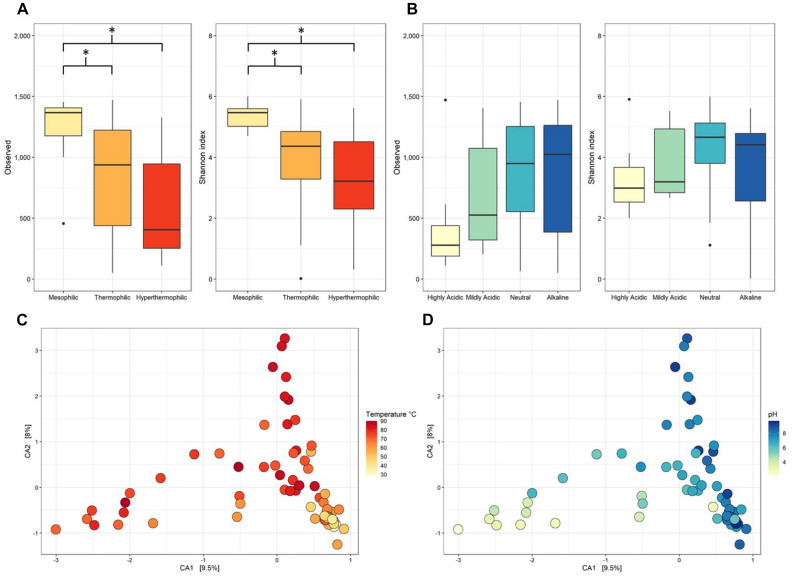
Observed taxa and Shannon Diversity metrics for the taxonomic profile assignment of metagenomic contigs at genus level classification. Taxonomy was assigned to metagenomic contigs using the Kraken2 standard database and contig abundances were calculated using Bracken. **(A)** α-diversity split by temperature categories, which were: mesophilic (<45.0°C), thermophilic (45.0–79.9°C), and hyperthermophilic (>80.0°C). **(B)** α-diversity split by pH categories, which were: highly acidic (<4.0), mildly acidic (4.0–5.9), neutral (6.0–7.9), and alkaline (>8.0). * denotes significance *p* < 0.05. Statistics calculated with pairwise comparisons and *p*-values adjusted by the Holm method. **(C,D)** Canonical correspondence analysis (CCA) of β-diversity calculated by Bray Curtis dissimilarity with points colored by sample temperature or pH, respectively.

Overall, mesophilic samples had the highest α-diversity followed by thermophilic, then hyperthermophilic samples. This result was supported by mesophilic samples being significantly higher than either thermophilic and hyperthermophilic samples for both observed taxa and Shannon Diversity (*p* < 0.001, Figures 2A,B and Supplementary Table 3 lists diversity metrics for each metagenome while Supplementary Table 4 contains statistical comparisons and *p*-values for each category tested). Several studies have found similar results of decreased α-diversity with increased temperature using 16S rRNA gene amplicon sequencing in hot springs (De León et al., 2013; Sharp et al., 2014; Lavrentyeva et al., 2018).

The α-diversity was lowest in the highly acidic samples but only for one of the diversity metrics tested. When pH of the samples increased, so did the α-diversity. However, the number of observed taxa for highly acidic metagenomes was not significant when compared to neutral or alkaline samples (*p* = 0.0726 and *p* = 0.137, respectively) and similarly, none of the pH categories were significantly different in terms of their Shannon Diversity (*p* > 0.05) with a *p*-value adjusted using the Holm method (Figures 2A,B and Supplementary Table 4). The α-diversity measured in our dataset aligns with the results of a 16S rRNA genes amplicon study of 925 New Zealand hot springs in which the highest diversity was found at 21.5°C and pH 6.4 with diversity decreasing toward higher temperatures and lower pH values (Power et al., 2018). The same study confirmed pH as the major driver of α-diversity in springs <70°C and that the pH of the system may affect the resultant nutrient and trace metal composition and availability. Consistently, a study on *Sulfolobus islandicus* populations in YNP hot springs attempted to elucidate effects of spring size, geographical location, and overall nutrients on microbial populations but pH was identified as the main driver of population diversity (Campbell et al., 2017).

Microbial community composition was affected by the temperature of the hot spring across all three temperature categories and for some of the pH categories. For β-diversity, Bray Curtis dissimilarity was plotted by canonical correspondence analysis (CCA) (Figures 2C,D) and the significance calculated by PERMANOVA. The β-diversity of all temperature categories were significantly different from each other in pairwise comparisons (*p* = 0.003). In contrast, the only pH comparisons that were significantly different were highly acidic samples to neutral and alkaline samples (*p* = 0.006) (Supplementary Table 4). As temperature increases and pH shifts to either end of the spectrum, microbial diversity has been observed to decrease in thermal systems (De León et al., 2013; Sharp et al., 2014; Menzel et al., 2015; Lavrentyeva et al., 2018; Power et al., 2018; Podar et al., 2020), an observation that is consistent with soils (Lauber et al., 2009). The notion that photosynthetic microorganisms are typically absent at temperatures >74°C due to the instability of pigments at such high temperatures explains the significance between thermophilic and hyperthermophilic communities. The microbial community in highly acidic sites was dominated by known acidophiles, specifically *Hydrogenobaculum*, *Sulfurihydrogenibium*, and *Sulfolobus*, which were the source of diversity compared to samples with pH > 6.0.

Many studies have analyzed hot spring microbial communities but were either limited by the availability of metagenomic datasets or focused on 16S rRNA gene amplicon short reads, which are limited in their taxonomic resolution and potential for functional profiling. Here, we have described the richness, evenness, and composition of 71 metagenomic assemblies. Temperature and pH ranges influenced the microbial communities and significant differences were observed for both α- and β-diversity. The α-diversity was not significant between thermophilic (45.0–79.9°C) and hyperthermophilic (>80.0°C) categories, however the microbial community analyzed by β-diversity revealed the composition of these temperatures to be significantly different (*p* = 0.003). In a previous 16S rRNA gene amplicon based study, pH was seen to be the main variable in community diversity below 70°C, with temperature becoming the main driver of diversity above this threshold (Power et al., 2018). Similarly, a non-linear trend of decreasing α-diversity was observed for several hot springs with a sharp drop in diversity above 80°C (Podar et al., 2020) and no significance in a temperature range of 50–80°C suggests the range of temperature or categories used for analysis is important to consider.

These results demonstrate the existence of unique microbial communities in physicochemically diverse conditions geothermal sites, suggesting that hot springs could provide a unique opportunity for novel CAZyme discovery.

### CAZyme Profile

To better understand the biomass degradation potential in each metagenome, CAZyme families related to cellulases, hemicellulases, and oligosaccharide-degrading enzymes were analyzed. The 71 metagenomes were examined with dbCAN2 for potential CAZymes, selecting only gene hits detected by at least two of the three methods utilized by dbCAN2 (Diamond, Hotpep, and HMMER). Two additional metagenomes, obtained from the P3 gut compartment of a *Nasutitermes corniger* termite (He et al., 2013; 2030936001) and a compost sample from the São Paulo Zoo (Martins et al., 2013; 2209111003), were included to compare metagenomes from geothermal features to environmental systems known for their high rates of plant biomass degradation. The *N. corniger* gut metagenome, an anaerobic sample, contained high relative abundances of cellulases and hemicellulases that degrade components of the termite’s wood-based diet (He et al., 2013). The aerobic compost metagenome was sampled from the São Paulo Zoo and contained enzymes for cellulose degradation along with additional CAZymes for lignin and pectin degradation (Martins et al., 2013).

**FIGURE 3:**
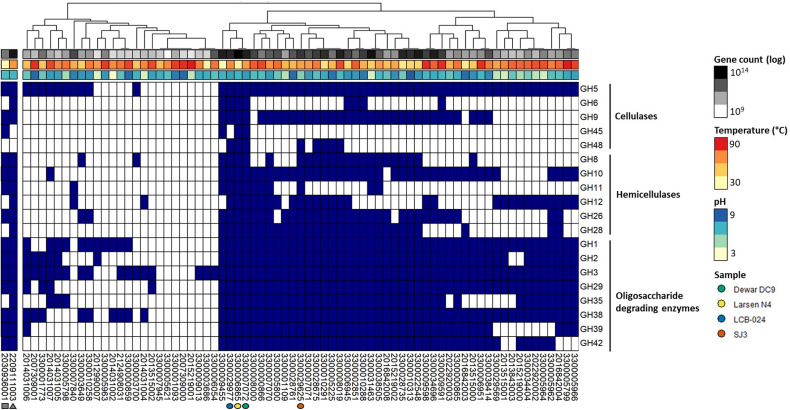
Heatmap showing presence-absence of specific CAZymes with predicted cellulase, hemicellulase, and oligosaccharide-degrading enzyme activities in the 71 metagenomes. Dark blue color represents the presence of a CAZyme gene sequence. Sample clustering was based on hierarchical cluster analysis of dissimilarity of the CAZyme presence-absence profile. Two metagenomes from non-hot spring lignocellulolytic environments are shown on the left and indicated with a gray square (2030936001—*Nasutitermes corniger* P3 gut compartment) and triangle (2209111003—São Paulo Zoo compost). Four hot spring metagenomes of particular interest to this study are indicated with colored circles. 3300029977—LCB-024—blue, 3300006865—Larsen N4—yellow, 3300007072—Dewar Creek DC9—green, and 3300029625—SJ3—orange.

**FIGURE 4:**
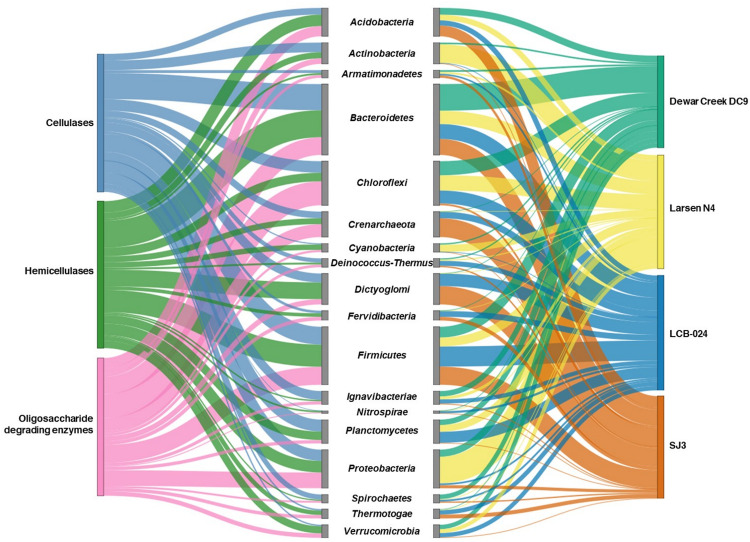
Sankey diagram illustrating taxonomic distribution of CAZyme-containing contigs and the hot spring metagenomes they originated from. Indicated on the left is the enzyme type determined by the most abundant Pfam domain for the most abundant glycosyl hydrolase family for contigs identified by dbCAN2 using the CAZy database (Pfam00150—GH5—Cellulase, Pfam00331—GH10—Hemicellulase, Pfam00933—GH3—Oligosaccharide-degrading enzymes). In the center is the taxonomic affiliation of the contigs determined by IMG. Taxonomically unclassified contigs were removed prior to plotting. Shown on the right is the hot spring metagenome location for the taxa. The height of the Sankey bars are assigned proportionately to the relative abundances of CAZyme-containing contigs and taxa.

The chemical nature and availability of carbohydrates pressures microbiome to adapt their CAZyme composition. For example, varying the feed or diet of ruminants resulted in differences in their microbiota and CAZyme families (He et al., 2013; Wong et al., 2017; Bohra et al., 2019). In addition to ruminants, soil (López-Mondéjar et al., 2020) and oil reservoir (Lewin et al., 2017) ecosystems have been well-studied in regards to CAZymes potential but studies on hot spring CAZymes are lacking. The termite gut and compost metagenomes were included in our analysis as controls of high potential CAZyme systems able to degrade plant biomass, encoding 17 and 14 CAZyme families, respectively; they were excluded from clustering. When examining CAZyme families by relative abundance, the two non-geothermal metagenomes have high relative abundance of GH3 and GH5 genes compared to all CAZymes (Supplementary Figure 1). These two glycosyl hydrolases are important CAZyme families for oligosaccharide and cellulose degrading activity, respectively. Figure 3 shows the presence or absence and clustering of CAZymes attributed to major enzyme families for cellulase, hemicellulase, and oligosaccharide degradation for the 71 hot spring metagenomes (Figure 3). CAZyme hits and corresponding IMG/M identification for all metagenomes are included in Supplementary Table 5. Three hot spring metagenomes that were also among the most deeply sequenced samples included in this study contained nearly all 20 CAZyme families, including the major families for cellulases, hemicellulases, and oligosaccharide-degrading enzymes. Two of these metagenomes, LCB-024 (3300029977, 69.4°C, pH 7.79; Lynes et al., in preparation) and Dewar Creek DC9 (3300007072, 64.7°C, pH 7.94), had been obtained from thermophilic, pH-neutral hot springs and contained 18 and 19 of the selected 20 CAZyme families, respectively. Dewar Creek DC9 is one of several samples collected from an interface between geothermal source water and soil. The third metagenome, Larsen N4, had been obtained from a mesophilic, pH-neutral geothermally affected sediment (3300006865, 33.1°C, pH 7.16) and contained 19 of the 20 CAZyme families. One additional metagenome from the mildly acidic hot spring SJ3 (3300029625, 61.9°C, pH 5.4, Colman et al., 2019) was included for further analysis to expand the range of spring conditions analyzed and contained 17 of the 20 CAZymes (Figure 4). We analyzed these metagenomes on a community-wide level to determine the overall biomass-degrading potential of geothermal sites with high CAZyme variability. All sequencing statistics are visualized in Figure 1 and are detailed in Supplementary Table 1.

These four metagenomes (Dewar Creek DC9, Larsen N4, LCB-024, and SJ3) contained a wide variety of biomass-degrading CAZymes and were further analyzed for the taxonomic affiliation of GH3, GH5, and GH10 containing contigs, which were the most abundant GH families for oligosaccharide-degrading enzymes, cellulases, and hemicellulases, respectively. Contig taxonomic identification was retrieved from IMG/M and plotted to show enzyme class and hot spring sample origin (Figure 4). Cellulase- and hemicellulase-containing contigs were assigned to fewer phyla (20 and 22 phyla, respectively) than oligosaccharide-degrading enzyme contigs (39 phyla), but when phyla >1% relative abundance were plotted, all enzyme types were similar in the number of phyla represented (Figure 4, full table of contig taxonomy and relative percentages in Supplementary Table 6).

The relative abundance of unclassified CAZyme contigs varied among the four metagenomes. Dewar Creek DC9 contained the largest amount of such contigs at 50.1% unable to be taxonomically classified in IMG/M, followed by SJ3 at 44.1% and then Larsen N4 and LCB-024 at 38.0 and 36.8%, respectively. This highlights the novel taxonomic diversity that remains to be described and explored within hot spring systems. With more genomic screening, databases will be able to identify and assign taxonomy to contigs more readily, as well as recognize additional CAZyme genes encoded on the contigs.

Highly abundant taxa, such as *Acidobacteria*, *Bacteroidetes*, *Chloroflexi*, and *Firmicutes* were common for all enzyme types while several low abundance species belonging to phyla *Aigarchaeota*, *Calescasmantes*, *Euryarchaeota*, and *Parcubacteria* were only linked to oligosaccharide degrading enzymes. In an earlier study of 5,123 sequenced bacterial genomes, 56% of genomes were found to contain genes associated with β-glucosides while 24% of the total genomes contained cellulases and β-glucosides (Berlemont and Martiny, 2013). Additionally, a comparative genomics study on 24 soil *Acidobacteria* isolate genomes found GH3 in all examined genomes while GH5, GH8, GH9, GH12, and GH44 were only found in select subdivisions of *Acidobacteria* (Eichorst et al., 2018).

The *Acidobacteria* CAZymes detected in the four metagenomes were identified to belong to cellulases, hemicellulases, and oligosaccharide-degrading enzymes. SJ3 had the largest relative abundance of *Acidobacteria* compared to the other hot springs, which could be attributed to the adaptation of *Acidobacteria* subfamily 1 to mildly acidic conditions (Jones et al., 2009; Campanharo et al., 2016). Additionally, various genera from *Acidobacteria* subfamily 1 have been shown to utilize cellulose and xylan as a growth substrate while other members of this family have not, demonstrating that further research on the biomass degradation potential of *Acidobacteria* is warranted (Kielak et al., 2016). *Actinobacteria* contigs contained a major portion of CAZymes for Larsen N4 with GH families in all three categories of enzymes. This abundance was greater in Larsen N4 compared to the other hot spring metagenomes which had higher temperatures. Larsen N4, with a temperature of 33.1°C, was expected to have a different population of potential biomass degraders than the other springs that are above 60°C as was also seen by the difference in taxonomic composition of its community. *Actinobacteria* enrichment cultures from lower temperature systems like compost (Wang et al., 2016) and soil (Anderson et al., 2012) have been shown to have diverse CAZyme repertoire and cellulose degrading capabilities.

CAZymes encoded by members of the *Bacteroidetes* encompass all three enzyme families and were present across all four analyzed metagenomes. *Bacteroidetes* are a prolific lineage for biomass degradation and are found in a variety of environments including soil (Taillefer et al., 2018), compost (Wang et al., 2016), rumen (Opdahl et al., 2018), and hot springs (Eichorst et al., 2013). Recently, a novel member of the *Bacteroidetes* with the ability to hydrolyze xylan was isolated using crystalline cellulose (Liu et al., 2020). In contrast, other *Bacteroidetes* from similar environments did not grow on crystalline cellulose but were part of late-stage enrichments suggesting soluble products as their growth substrate (Eichorst et al., 2013). The variability of biomass degradation potential within the *Bacteroidetes* highlights this phylum-level lineage as a promising target for the identification of novel CAZymes in future research. In a recent study, proteomics was used to examine the enzymatic activity of CAZymes of two species grown on both cellulose and pectin. Expression of endoglucanases and β-glucosidases varied between the two and pending time of incubation (Taillefer et al., 2018).

Contigs attributed to *Chloroflexi* contained many oligosaccharide-degrading enzymes. Members of the *Chloroflexi* have been enriched and isolated from biomass-rich systems (e.g., wastewater and sludge samples) and had been seen in high abundances in late stage enrichments (Ishii et al., 2008), suggesting that some *Chloroflexi* are able to scavenge the breakdown products of cellulose using oligosaccharide-degrading enzymes. The decreased photosynthetic temperature limit under acidic conditions (Boyd et al., 2012; Inskeep et al., 2013b) could be one explanation for the decreased abundance of *Chloroflexi* in SJ3 relative to the other springs as many taxa of *Chloroflexi* from hot springs have phototrophic metabolisms (Klatt et al., 2011, 2013). We observed *Dictyoglomus* in the LCB-024 and SJ3 metagenomes with a majority of their contigs being assigned to contain hemicellulases. *Dictyoglomus* isolates have been shown to produce multiple xylanases (Mathrani and Ahring, 1992) and have the ability to grow on a variety of carbon polymers (Bacterium et al., 1985; Brumm et al., 2016). Interestingly, certain *Dictyoglomus* also encode glycosyl hydrolases with both cellulase and hemicellulase activity and are able to degrade carboxymethyl cellulose and mannans (Fusco et al., 2018). A broad pH growth range (pH 5–9) and high temperature optimum (72–80°C) with broad cellulase and hemicellulase activity highlights *Dictyoglomus* as an attractive organism for industrial lignocellulose degradation. Out of the 18 phyla present at >1% abundance in the four metagenomes, only *Fervidibacteria* currently lacks an isolated representative. However, *Fervidibacteria* has conflicting reports of biomass degradation potential. On the one hand, members of the *Fervidibacteria* have been enriched from a switchgrass inoculum (Peacock et al., 2013); on the other hand, hot spring (FS5, 74°C, pH 8.2) *Fervidibacteria* exhibited decreased translational activity in the presence of cellobiose or cellulose as compared to when these substrates were absent (Reichart et al., 2020).

*Firmicutes* are a high priority target for biomass conversion due to consolidated bioprocessing (CBP) of lignocellulose occurring in a single organism without the need for pretreatment and some of the most thermophilic cellulolytic organisms identified so far are within this phylum. *Firmicutes* contigs were abundant in the four metagenomes and contained genomic potential for all three enzyme types. The class *Clostridia* is anaerobic and can utilize a multi-enzyme structure called a cellulosome to stay bound to cellulose during degradation. The sugars cleaved from cellulose can then be fermented by *Caldicellulosiruptor thermophilum* producing lactate, ethanol, and acetate as end-products (Yang et al., 2009). *Caldicellulosiruptor* has been a target of metabolic engineering to increase rates of conversion of plant biomass to ethanol due to their potential for CBP. When Kim et al. (2018) expressed a cellobiose phosphorylase in *Caldicellulosiruptor bescii*, inhibition of cellulases by cellobiose was decreased, which in turn increased the cellulolytic activity. Chung et al. (2014) transformed a strain of *C. bescii* lacking lactate dehydrogenase to contain a plasmid-prone acetaldehyde/alcohol dehydrogenase gene to produce over 14 mM of ethanol from a 1% cellobiose solution compared to no ethanol production from the wild type strain. Another *Firmicutes* consortium enriched from a hot spring sample was able to degrade a variety of substrates and produced ethanol. This led to a shift in community composition over a 7-day incubation period, suggesting synergism and specialization of individual community members to specific steps during the process (Zhao et al., 2014). While a majority of the contigs remain taxonomically unclassified in IMG/M, many of the detected taxa are linked to species with biomass-degrading activity. Together, these results demonstrate the diversity of CAZyme-containing bacteria in hot springs and highlight the attractiveness of these extreme ecosystems for future biotechnological research.

Owing mainly to the lack in archaeal isolates (Baker et al., 2020; Sun et al., 2020), archaeal CAZymes have drastically lagged characterization of their bacterial counterparts. At the time of publication, the CAZy database contained 18,171 bacterial genomes and 392 archaeal genomes. The majority of archaea capable of cellulose degradation are affiliated to *Crenarchaeota* and *Euryarchaeota*, which also contain the majority of archaeal isolates, and have optimal temperatures ranging from 80 to 115°C when grown on cellulose (Suleiman et al., 2020). Isolates and enriched communities of *Crenarchaeota* have been recovered from several hyperthermophilic sources, including terrestrial hot springs (Perevalova et al., 2010; Graham et al., 2011; Zayulina et al., 2020). While we did not recover CAZyme containing contigs affiliated with *Euryarchaeota*, crenarchaeotal contigs were detected in LCB-024 and SJ3, with the latter hot spring containing more such contigs. SJ3 contigs were affiliated to genera *Caldivirga*, *Ignisphaera*, and *Thermofilum* and contained all three enzyme types. *Crenarchaeotal* contigs from LCB-024 were affiliated to genera *Ignisphaera*, *Thermofilum*, and *Thermosphaera* and encoded cellulases and oligosaccharide degrading enzymes. While most of these crenarchaeotal genera have a broad growth range of 60–98°C and are adapted to circumneutral pH (Huber et al., 1998; Niederberger et al., 2006; Toshchakov et al., 2015), *Caldivirga* isolates grow optimally at pH 4 (Itoh et al., 1999). These observations are consistent with a higher abundance of *Caldivirga*-associated contigs in the metagenome of the mildly acidic SJ3 site.

While this study focused on glycosyl hydrolases for biomass degradation, the output from dbCAN2 also included multiple hits corresponding to pectinases, peroxidases, and laccases that should also be examined for potential novel targets of lignin, and other recalcitrant biomass degradation of biotechnological impact. Carbohydrate-binding modules (CBM) for binding domains also provide information about the proximity of organisms to biomass for degradation as they would be bound to carbohydrate particles. The catalytic domains for lignin degradation are classified into several auxiliary activity (AA) families that have not been analyzed here. In addition, carboxyl esterase (CE) families are an alternative way to cleave bonds composing lignocellulosic biomass. One limitation of our study is that it did not examine the presence of polysaccharide utilization loci (e.g., PUL and cellulosome loci via dbCAN-PUL; Ausland et al., 2021); the analyses of these enzyme families were outside the scope of this work. However, the broad diversity in geochemistry and microbiology of hot springs and results reported herein suggest that geothermal sites are a promising source for future biotechnological applications. Another limitation is that the metagenomes investigated herein were generated over 13 years (2007–2020). Unfortunately, this made certain comparisons difficult because datasets were generated using dramatically different sequencing technologies and pipelines; this variation sometimes prohibited us from making as clear recommendation for future research into hot spring CAZymes as we initially hoped to make.

### Outlook

Hot springs are promising ecosystems for the discovery of biomass-degrading enzymes with high potential for unclassified or novel enzyme activities. The unique physicochemical extremes of thermal features make them an ideal testbed for identifying the next generation of highly lignocellulolytic organisms and their enzymes. In this study, we analyzed 71 metagenomes with a focus on four datasets from hot springs with mildly acidic to neutral pH. While these four metagenomes were among the most deeply sequenced, we stress that further bioprospecting of hot springs should occur across all temperature and pH ranges, given enough sequencing coverage. At this point, no particular geochemical niche (e.g., specific range of temperature or pH) emerged as exceptional site for investigations into biomass degradation; we thus recommend being as inclusive as possible in future enzyme discovery studies. Improvements in genomic library creation and high throughput sequencing have provided an opportunity to query communities from low biomass hot spring samples at greater depth and coverage for metagenomics (DeCastro et al., 2016). Challenging to cultivate organisms can now be accessed via single cell genomics (Rinke et al., 2014; Alteio et al., 2020) and metagenomics and genes of high interest can be expressed in a wider range of amenable hosts (Girfoglio et al., 2012; Wang et al., 2019) for lab characterization via functional metagenomics (Milshteyn et al., 2014; Van Der Helm et al., 2018). In order to screen the large diversity of yet uncharacterized CAZymes found in hot springs and other ecosystems, we recommend to combine the above approaches with high through-put screening tools, such as microfluidics-based functional enzyme assays, capable of screening thousands to tens of thousands of enzymes in parallel (Brouzes et al., 2009; Leung et al., 2012; Terekhov et al., 2017, 2018).

To help advance future bioprospecting studies, we call for considerable improvements to the minimum metadata collected, especially for geochemical parameters of sampling locations. More metadata would allow to contextualize and more directly compare different metagenome datasets as well as provide opportunities to identify possible trends of microbial community composition and function across various sampling efforts and studies. Several studies have attempted to test whetherthe microbial community composition of hot springs can be predicted based on geochemical parameters (Inskeep et al., 2013b; Campbell et al., 2017; Power et al., 2018; Kochetkova et al., 2020) but a lack in contextual data make comparison across datasets obtained by different laboratories challenging. Other than pH, temperature, location, and date of sample acquisition, which already need to be reported if a dataset is to be added to IMG/M, valuable parameters to report in future studies should include dissolved oxygen, salinity, sulfide, total and dissolved (in)organic carbon and nitrogen, as well as trace metal availability. Similarly, we were unable to comment on the presence of plant biomass (e.g., tree trunks, pinecones, grass) in specific features. While photos were available for most hot springs in primary publications or online databases, the exact location of DNA sample acquisition was typically not included; because of this, it was impossible to correlate the presence of plant biomass with the presence of cellulose degraders.

The majority of biomass-degrading enzymes in hot springs remains to be discovered and biochemically characterized. Metagenome-based discovery studies of CAZymes will benefit from further exploration and expansion of databases to link functional activity to genomic predictions. With continued focus on underexplored environments and taxa, novel or higher efficiency CAZymes will be discovered to aid in the industrial processing of lignocellulosic biomass for more efficient biofuel conversion.

## Data Availability Statement

The original contributions presented in the study are included in the article/Supplementary Material, further inquiries can be directed to the corresponding author/s.

## Author Contributions

NJR, TW, and RH conceptualized and designed the study. NJR and RMB performed the data analyses. NJR drafted the manuscript. All authors edited the manuscript.

## Disclaimer

All opinions expressed in this manuscript are the author’s and do not necessarily reflect the policies and views of DOE, ORAU, or ORISE.

## Conflict of Interest

The authors declare that the research was conducted in the absence of any commercial or financial relationships that could be construed as a potential conflict of interest.

## Footnotes

1 https://ethanolrfa.org

2 http://rcn.montana.edu/Default.aspx

3 http://www.cazy.org/
