# Supplementary figures and images for: High Potential for Biomass-Degrading Enzymes Revealed by Hot Spring Metagenomics
Source: Front Microbiol. 2021 Apr 21;12:668238. doi: 10.3389/fmicb.2021.668238 (PMC8098120; doi:10.3389/fmicb.2021.668238)

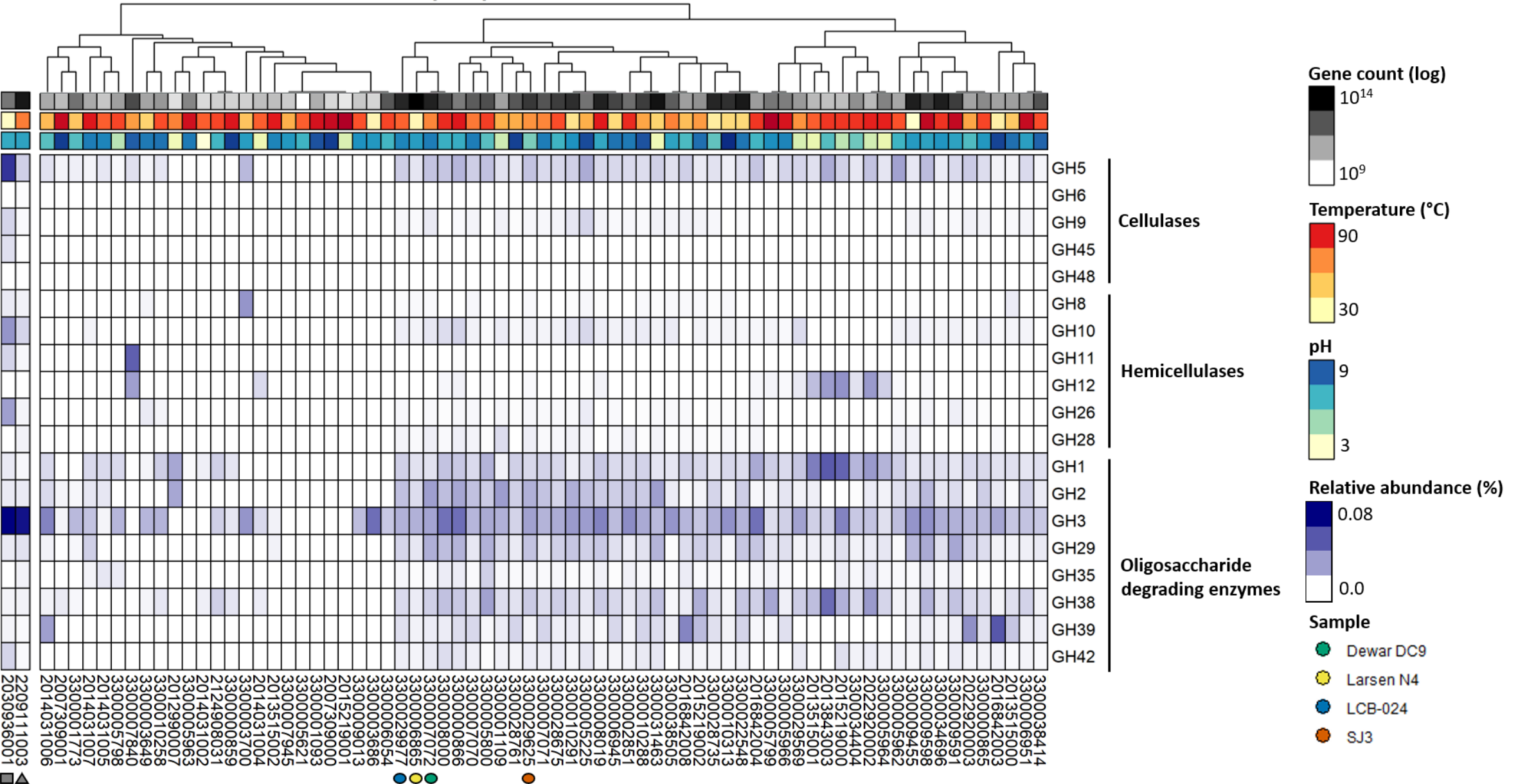

Supplement: Supplementary file 1 [file Image_1.TIFF]
