# Supplementary material for: High Potential for Biomass-Degrading Enzymes Revealed by Hot Spring Metagenomics
Source: Front Microbiol. 2021 Apr 21;12:668238. doi: 10.3389/fmicb.2021.668238 (PMC8098120; doi:10.3389/fmicb.2021.668238)
Supplement: Supplementary file 3 [file Table_2.DOCX]

**Supplementary Tables Legends**

Table 1. Metagenome metadata. This file contains the TaxonOID for correct identification to the IMG database along with pertinent metadata on sample collection and any publication information.

Table 2. Taxonomic profile at the genus level from Kraken2/Bracken for all metagenomes.

Table 3. Output of α-diversity results for all metagenome taxonomic profile. Taxonomy of the metagenomic contigs was assigned using Kraken2 and the relative abundance calculated with Bracken. Diversity metrics were measured using Phyloseq R package.

Table 4. Pairwise comparisons for α- and β-diversity for the taxonomic profile of temperature and pH categories. The α-diversity compared observed taxa and Shannon diversity metrics using the lme4 R package. The β-diversity compared Bray Curtis dissimilarity of taxonomic composition using the vegan R package. The p values were adjusted for pairwise comparisons using the Holm method using the base R stats function p.adjust().

Table 5. CAZyme hits corresponding to IMG/M gene IDs for all IMG/M taxon OIDs. Only CAZyme genes with hits to two or more of dbCAN2 tools are listed and used within the analysis.

Table 6. Sankey links used for generation of Figure 4. All taxa present in the enzyme output included in this file.

**Supplementary Figure Legend**

Figure 1. Relative abundance of CAZyme families out of total CAZyme gene hits. Specifically, 19 CAZymes with predicted cellulase, hemicellulase, and oligosaccharide degrading enzyme activities for 71 hot spring metagenomes and two additional non-hot spring metagenomes from cellulolytic sources. Sample clustering was kept consistent based on the dendrogram output from main text Figure 3. Symbols are as follows: grey square (2030936001 - *Nasutitermes corniger* P3 gut compartment), grey triangle (2209111003 - São Paulo Zoo compost), blue circle (3300029977 - LCB-024), yellow circle (3300006865 - Larsen N4), green circle (3300007072 - Dewar Creek DC9), and orange circle (3300029625 - SJ3). We caution the reader that direct comparisons of CAZyme abundances between features cannot be made due to the different sequencing technologies used during the 13 year timespan.
